# CARE-NS, a research strategy for neurosyphilis

**DOI:** 10.3389/fmed.2022.1040133

**Published:** 2023-01-06

**Authors:** Fang-Zhi Du, Xu Zhang, Rui-Li Zhang, Qian-Qiu Wang

**Affiliations:** ^1^Institute of Dermatology, Chinese Academy of Medical Sciences and Peking Union Medical College, National Center for STD Control, China Centers for Disease Control and Prevention, Nanjing, China; ^2^Department of Dermatology, The Second Affiliated Hospital of Nanjing Medical University, Nanjing, China

**Keywords:** neurosyphilis, CARE-NS strategy, clinical management, prevention and control, epidemiology

## Abstract

Neurosyphilis is a major clinical manifestation of syphilis. In recent years, an increase in neurosyphilis cases has been reported in many countries. The overall incidence of neurosyphilis remains unknown, and there is a lack of understanding of the disease pathogenesis, which hampers clinical management, development of prevention strategies, and control. This article proposes the CARE-NS research strategy to enhance the clinical management of neurosyphilis, which consists of six key features: comprehensive management including multidisciplinary treatment (C), alleviating neurological impairment and sequelae (A), risk factors and clinical epidemiology (R), etiology and pathogenesis (E), new diagnostic indicators and strategies (N), and social impact and cost-effectiveness analysis (S).

## Introduction

Neurosyphilis, caused by invasion of the central nervous system (CNS) by *Treponema pallidum* subsp. *pallidum* (*T. pallidum*), has puzzled dermatologists, neurologists, and psychiatrists for over two centuries because of its complex and non-specific clinical manifestations and the lack of a standard diagnostic criterion. Neurosyphilis can lead to irreversible damage to the CNS and even death if patients are not administered treatment early. Since 2000, following the resurgence of syphilis in low- and middle-income countries and in some populations in developed countries (1), patients presenting with symptoms of neurosyphilis have been more frequently reported by clinicians. The incidence of neurosyphilis remains unknown in most countries due to a lack of comprehensive syphilis surveillance programs (e.g., in China). Subsequently, few studies have reported the prevalence of neurosyphilis in any context. For instance, the proportion of neurosyphilis cases in early-stage syphilis patients from 2009 to 2015 was 1.8% on average in 10 states in the United States (2). In Australia, the annual incidence of neurosyphilis between 2007 and 2016 was 2.47 per 100,000 (3). Increases in the incidence of neurosyphilis in some countries have been reported in recent years. For example, in the Canadian province of British Columbia, the incidence increased from 0.03 per 100,000 in 1992 to 0.8 per 100,000 in 2012, representing a 27-fold increase across 11 years (4). From 1999 to 2010, neurosyphilis accounted for approximately 10–13% of total syphilis cases in the Netherlands, with an annual average incidence of 0.47 per 100,000 (5). The reported incidence of neurosyphilis in Guangdong Province, China, increased from 0.21 per 100,000 in 2009 to 0.31 per 100,000 in 2014, with an annual increase of 8.1% (6). A recent survey found that from 2017 to 2019, the average annual incidence of neurosyphilis in five cities was 0.31, 0.48, and 0.68 per 100,000, respectively, with an average annual growth of 48.11% (7). These data demonstrate a trend of rapidly increasing neurosyphilis cases. As cases are relatively rare compared with those of syphilis, the prevention and control of neurosyphilis has been neglected in many countries. On 18 July 2022, the WHO issued the “*Global health sector strategies on, respectively, HIV, viral hepatitis, and sexually transmitted infections for the period 2022–2030*,” which suggested that early detection and treatment of sexually transmitted diseases should be strengthened to prevent serious complications or sequelae (8). Despite this, a poor understanding of neurosyphilis pathogenesis, the lack of an effective vaccine, inconsistent diagnostic criteria in many countries, difficulties in early detection, common occurrence of treatment failure, and difficulties in evaluating prognosis remain obstacles to the prevention and control of neurosyphilis worldwide.

Therefore, the CARE-NS research strategy was proposed in 2019 by the National Center for sexual transmitted disease (STD) Control, Chinese Centers for Disease Control and Prevention. CARE-NS aims to resolve key problems and obstacles in the clinical management and research of neurosyphilis. The research strategy will provide important evidence to develop strategies for the prevention and control of neurosyphilis.

## CARE-NS strategy

### Comprehensive management including multidisciplinary treatment (C)

Neurosyphilis can cause multiple organ damage. Early injury occurs within the first 2 years and predominantly affects the mesenchymal region of the brain, such as the meninges and surrounding blood vessels, leading to meningitis and spinal membrane damage. Thereafter, late forms of injury occur between years and decades after primary infection and affect the parenchymal region of the brain and spinal cord, which manifests as cerebral infarction, general paresis, and tabes dorsalis (8). Additional symptoms of neurosyphilis include vision and hearing loss, as well as ocular and otic inflammation. As a result, the diagnosis and treatment of neurosyphilis span multiple clinical departments (9). It is therefore necessary to establish standardized diagnostic criterion and personalized treatment strategies with multidisciplinary experts.

In addition, developing a clinical pathway for neurosyphilis, will provide measurable improvements in patient outcomes, as well as fundamentally complements clinical advances in clinical settings. Although the clinical management of neurosyphilis is complex, screening strategies for this condition are clear. Laboratory and imaging examinations are readily available, diagnostic criteria are applicable, and treatment regimens are clear, creating a basis for establishing a reasonable clinical pathway for neurosyphilis. Furthermore, strengthening the training of clinicians from other departments in the diagnosis and treatment of neurosyphilis is required to enhance their understanding of the diagnostic criteria, treatment regimens, and clinical pathway.

### Alleviating neurological impairment and sequelae (A)

Previous studies have demonstrated significant improvement of clinical symptoms and recovery of abnormal cerebrospinal fluid (CSF) indicators after treatment of early neurosyphilis compared with that of late neurosyphilis (8). Patients with meningitis and intracranial gumma can completely recover after adequate anti-syphilis treatment; however, while the meningeal symptoms and signs of meningovascular syphilis can disappear after treatment, symptoms of stroke sequelae may persist. The progression of neurosyphilis can also be halted by anti-syphilis treatment in patients with general paresis or tabes dorsalis, but the symptoms of dementia or sensory ataxia often do not improve (1, 8). In addition, some early clinical manifestations may be associated with the prognosis of neurosyphilis. Ozturk-Engin et al. (10) found that reported headaches in syphilis patients are beneficial for the early detection of neurosyphilis and correlate with a positive prognosis, while patients with diplopia symptoms are significantly associated with adverse prognoses, such as neurological sequelae and cognitive impairment. Therefore, early diagnosis of neurosyphilis, timely anti-syphilis treatment, early involvement of neurologists, and exploration of predictors of prognosis to provide appropriate interventions are essential.

The current treatment regimen recommended by the National Guidelines is mainly anti-syphilis treatment, which aims to kill *T. pallidum* and avoid sustained neurological damage. However, there is a lack of *in vitro* studies and large prospective clinical trials to evaluate the definitive efficacy of penicillin G and other recommended drugs, as the recommended regimen is predominantly based on the pharmacokinetics of available drugs, laboratory tests, biological plausibility, expert opinion, case studies, and clinical experience (11). Recently, two retrospective studies found no significant difference in the overall efficacy of ceftriaxone versus aqueous penicillin treatment and no difference in the rates of clinical and serological response when patients were treated with procaine G penicillin versus doxycycline (12, 13). Furthermore, a prospective randomized controlled trial demonstrated no difference in symptom recovery between neurosyphilis patients with psychiatric symptoms who were treated with either ceftriaxone or aqueous penicillin (14). However, there is a lack of evidence for the optimal dose and duration of treatment for all drugs except penicillin G (15). On the other hand, neurological damage caused by invasion of *T. pallidum* in the CNS requires nutrition and nerve repair drugs for improvement. Unfortunately, there is no recommended regimen for the use of neuropsychiatric drugs for dealing with neurological damage and sequelae. Therefore, multicenter trials are required to identify the optimal regimens for antimicrobial drugs and neuropsychiatric drugs. These studies will provide evidence-based medical data to develop comprehensive treatment regimens for repairing nervous system damage and preventing sequelae.

### Risk factors and clinical epidemiology (R)

Neurosyphilis is reported in cases of tertiary syphilis. From 2014 to 2019, the number of reported cases and the incidence of tertiary syphilis increased by 1.98 and 1.61% in China annually (16), respectively, suggesting that the incidence of neurosyphilis shows a slow upward trend. Studies have demonstrated that older males and patients with serum rapid plasma reagin (RPR) titers ≥1:32 are high-risk groups among HIV-negative asymptomatic neurosyphilis patients (17, 18). The proportion of neurosyphilis was also higher in serofast syphilis patients and those coinfected with HIV (19). Furthermore, the risk of neurosyphilis correlates with the stage of syphilis infection, whether patients have received previous treatment for syphilis, whether they present with neurological symptoms, their CD4+ T-cell counts, neutrophil to lymphocyte ratios, and the elevation of white blood cells and protein indices in the CSF (20–22). These risk factors provide evidence for screening in susceptible populations for early detection of neurosyphilis.

Clinical epidemiology studies can evaluate the clinical characteristics, diagnostic methods and strategies, therapeutic efficacy, and prognosis of neurosyphilis to provide evidence for improved clinical management and individualized treatment. A clinical epidemiological analysis of 117 neurosyphilis patients conducted by Chen et al. (23) found that the onset age, CSF protein concentration, positive rate of RPR, and rate of abnormal imaging examination were significantly higher in patients with cerebral parenchymal neurosyphilis than in asymptomatic or mesenchymal neurosyphilis patients, which indicated that the different clinical types apply different diagnostic strategies. Li et al. (24) found that elevated serum RPR titers, CSF protein concentrations, and CSF RPR titers may indicate the development of neurosyphilis and the aggravation of neurological symptoms. Martínez-Ayala et al. (25) found that neurological symptoms, particularly headache, were predictors of neurosyphilis in people with HIV irrespective of their viral load and lymphocyte CD4+ T-cell count in late latent syphilis, which provided evidence for early detection of neurosyphilis in HIV-infected patients. Regarding molecular epidemiology, Christina et al. (26) found that the *T. pallidum* 14d/f genotype was associated with susceptibility to neurosyphilis. In 2012, Dai et al. (27) conducted a study on the molecular typing of syphilis in a Chinese population, which showed that 14d/f and 19d/c genotypes were found in neurosyphilis cases, but no significant association with the risk of neurosyphilis could be discerned due to a limited sample size. Different genotypes of *T. pallidum* may present with different virulence profiles and be associated with different clinical manifestations. Molecular epidemiological studies can identify the dominant genotypes of *T. pallidum* in different regions and provide evidence for tracing transmission networks, which may guide clinical intervention. However, there is a paucity of neurosyphilis clinical and molecular epidemiology studies, especially considering the analysis of clinical efficacy and prognosis and the analysis of neurosyphilis prevalence at the molecular level. Future clinical and molecular epidemiological studies should leverage large sample sizes to provide evidence for improving the clinical management and precision treatment of neurosyphilis.

### Etiology and pathogenesis (E)

Previous studies have demonstrated that *T. pallidum* can translocate across the blood-brain barrier (BBB) *via* intercellular connectivity. Recent studies have shown that inflammatory factors play an important role in regulating junctions between BBB cells. For example, the cytokine IL-17 compromises the connection between tight junction occludins and activates endothelial contraction to increase BBB permeability (28). Toll-like receptors (TLRs) and interferon γ (IFN-γ) receptors synergistically stimulate the expression of nitric oxide synthase in microglia, thereby inducing neurotoxicity and increasing BBB permeability (29). CNS invasion by *T. pallidum* promotes increased CSF levels of IL-17 and IFN-γ, indicating a potential role in BBB disruption (30). The *T. pallidum* adhesion protein Tp0751 may affect the expression of tight junction proteins by inducing apoptosis and promoting secretion of the inflammatory cytokine IL-6, which may facilitate BBB translocation (31). *T. pallidum* also upregulates the expression of chemokine (C-X-C motif) ligand 6 (CXCL6), CXCL8, and CXCL10 in human microvascular endothelial cells (HBMECs), which enhances the chemotaxis of HBMECs on HL-60 cells (32).

After CNS invasion, *T. pallidum* can directly damage blood vessels and cause vascular inflammation, tissue damage, and the secretion of inflammatory cytokines *in vivo*. The pathogenic proteins of *T. pallidum* and the subsequent immune regulation of B and T cells may be involved in these pathophysiological processes. Tp0751 can promote adhesion of *T. pallidum* to host cells (33), Tp0965 can activate endothelial cells to increase their permeability (34), and Tp0326 promotes the metabolic proliferation of *T. pallidum* in infected host cells (35). The mechanism underpinning the role of these proteins in CNS injury remains unclear. Drago et al. (36) found that the levels of CXCL13 and IFN-γ were increased in the CSF of neurosyphilis patients. CXCL13 is a chemokine induced by B cells, and detection of CXCL13 in the CSF indicates that intrathecal antibodies can be synthesized to counter antigens. Other studies have found that the number of Treg cells in the CSF is reduced such that they are not sufficient to inhibit T-cell-mediated inflammation and subsequent injury to the meninges, brain parenchyma, spinal cord, and other tissues (37). Recent studies have also found that *lncRNA-ENST00000421645* promotes T-cell apoptosis in patients with neurosyphilis by mediating IFN-γ production through interaction with PCM1 protein (38). An additional study showed that *T. pallidum* can adhere to the host cell surface and capillary lining through mucopolysaccharide enzymes, which destroy mucopolysaccharide-rich blood vessels. This promotes necrosis of the inner and outer blood vessel membranes, leading to hemorrhage, thrombosis, and ischemic infarction, which further aggravates the surrounding tissue and CNS damage due to the inflammatory response induced by inflammatory cytokines and inflammatory cells that leak out of the blood vessels (39).

The autoimmune CNS response, including activation of microglia, can be beneficial for the survival of neurons and can induce conducting intercellular immune regulation. The mechanism of microglial activation and functional changes during neuronal injury in neurosyphilis requires further clarification. Host genetics may also influence susceptibility to neurosyphilis. Polymorphisms in TLR1 (1805 T→G), TLR2 (2258 G→A), and TLR6 (745 C→T), in addition to interleukin (IL) 10-promoter polymorphisms (1084 G→A and 592 C→A), are associated with susceptibility to neurosyphilis (40, 41).

The pathogenetic mechanism of neurosyphilis remains unclear. Therefore, the etiological basis of the different clinical manifestations, pathogens, and host factors related to the pathogenesis and their interaction need further systematic research. Establishing methods for the long-term culture of *T. pallidum in vitro* would be beneficial to create breakthroughs in this field.

### New diagnostic indicators and strategies (N)

The recommended laboratory diagnostic indices for neurosyphilis have limitations for guiding clinicians in diagnosis, treatment, and follow-up. At present, CSF venereal disease research laboratory test (VDRL) is the gold standard for specificity in the absence of blood contamination, but its sensitivity is still a topic of debate, its operation is complex, and the availability of reagents is poor (42). Fluorescent treponemal antibody absorption test (FTA-ABS) is highly sensitive, but specificity is poor, and it suffers from poor reagent availability (43). There is thus a clear need to identify suitable diagnostic biomarkers. PCR detection of *T. pallidum* in the CSF has been temporarily discontinued due to low sensitivity and specificity (44). Increased concentrations of the chemokines CXCL13, CXCL8, and CXCL10 in the CSF or their CSF/serum ratio, particularly in the case of CXCL13, can predict the occurrence of neurosyphilis (45). In addition, levels of macrophage migration inhibition factor in the CSF, peripheral blood CD8+IFN-γ+ cells, levels of serum IL-26, and antibody index (AI) for intrathecal synthesis of specific anti-treponemal IgG were predictive of neurosyphilis (46–49). Furthermore, abnormal expression of mir-590-5p, mir-570-3p, mir-570-5p, and mir-21-5p in serum and CSF is another potential biomarker of neurosyphilis (50). However, none of these biomarkers are used for clinical diagnosis, as their specificity and sensitivity have not been evaluated, and they lack validation and evaluation with large sample sizes. Among these biomarkers, CXCL13 has been used as an auxiliary reference for the diagnosis of neurosyphilis in the latest guidelines in China (9) but still falls short for application in clinical diagnosis.

Follow-up visits of neurosyphilis patients are required every 3–6 months and last for more than 3 years after treatment. Lumbar puncture is an invasive examination, and patients are often non-cooperative, leading to low follow-up rates. Furthermore, white blood cell (WBC) counts in the CSF are currently used as the main laboratory reference index in follow-ups but vary greatly among patients (51); thus, its sensitivity and accuracy as a prognostic indicator is insufficient. Therefore, validation of novel biomarkers with higher sensitivity and accuracy to establish diagnostic criteria and follow-up requirements for neurosyphilis should be a priority. It remains a major challenge to establish diagnostic criteria and follow-up requirements for neurosyphilis based on high-quality evidence-based medicine.

### Social impact and cost-effectiveness analysis (S)

Severe neuropsychiatric symptoms are common in neurosyphilis patients who do not quickly receive diagnosis and treatment, resulting in a large social burden. A survey on the burden of neurosyphilis in five cities in China from 2017 to 2019 showed that the disability-adjusted life years (DALYs) were 323.35, 455.42, and 630.92 person-years in 2017, 2018, and 2019, respectively, and the DALY rates were 1.66/100,000, 2.30/100,000, and 3.13/100,000, respectively, with an annual growth of 37.3%. The average hospitalization cost for neurosyphilis patients was 18,136.60 yuan, and the average loss was 278,500 yuan (7). The total hospitalization cost and indirect economic loss increased year by year. These data indicate a high burden of neurosyphilis in China. Currently, the lack of psychosocial factors and cost-benefit analysis for neurosyphilis hamper a complete assessment of the social impact of this disease and cost to the health services. At present, social impact and cost-benefit analyses mainly focus on prenatal screening for gestational syphilis (52) and syphilis screening for high-risk groups, such as men who have sex with men (53). An analysis of screening for serofast syphilis patients in Shenzhen showed a low cost with a cost-effectiveness ratio of 1:24.97 (54). Unfortunately, there are no reports of disease burden and the cost-benefit analysis of screening for neurosyphilis internationally. Further research on the social and economic cost of neurosyphilis is required to accurately evaluate the disease burden and cost-effectiveness of health care services for neurosyphilis. Publicity and education, consultation, screening, referral, treatment, and follow-up will provide evidence for the establishment of effective strategies for the prevention and control of neurosyphilis.

## Conclusion

The CARE-NS research strategy for neurosyphilis will address questions as follows: (1) Developing a multidisciplinary treatment model and clinical pathway for standardized clinical management. (2) High-quality clinical trials design, optimization, and development to improve the treatment guideline. (3) Revealing the risk factors, clinical, and molecular epidemiological characteristics of neurosyphilis for precision medicine. (4) Definite diagnostic criterion and prognostic predictors to the early detection and improvement of prognosis. (5) Clarifying etiology and pathogenesis of neurosyphilis. Widening national and international participation in neurosyphilis research will improve the control of neurosyphilis worldwide. We anticipate that the research outlined in this strategy will provide powerful scientific evidence for the prevention and control of neurosyphilis.

## Data availability statement

The original contributions presented in this study are included in the article/supplementary material, further inquiries can be directed to the corresponding authors.

## Author contributions

F-ZD wrote the draft. Q-QW and R-LZ revised the manuscript. F-ZD and XZ searched the literature. All authors contributed to the article and approved the submitted version.
